# Dynamic Patterns of Mammalian Mitochondrial DNA Replication Uncovered Using SSiNGLe-5′ES

**DOI:** 10.3390/ijms24119711

**Published:** 2023-06-03

**Authors:** Dongyang Xu, Lingcong Luo, Yu Huang, Meng Lu, Lu Tang, Yong Diao, Philipp Kapranov

**Affiliations:** 1 Institute of Genomics, School of Medicine, Huaqiao University, 668 Jimei Road, Xiamen 361021, China; xudongyang@hqu.edu.cn (D.X.);; 2 State Key Laboratory of Cellular Stress Biology, School of Life Sciences, Xiamen University, Xiamen 361102, China

**Keywords:** mitochondria, mitochondrial genome, origin of DNA replication, SSiNGLe-5′ES

## Abstract

The proper replication of mitochondrial DNA is key to the maintenance of this crucial organelle. Multiple studies aimed at understanding the mechanisms of replication of the mitochondrial genome have been conducted in the past several decades; however, while highly informative, they were conducted using relatively low-sensitivity techniques. Here, we established a high-throughput approach based on next-generation sequencing to identify replication start sites with nucleotide-level resolution and applied it to the genome of mitochondria from different human and mouse cell types. We found complex and highly reproducible patterns of mitochondrial initiation sites, both previously annotated and newly discovered in this work, that showed differences among different cell types and species. These results suggest that the patterns of the replication initiation sites are dynamic and might reflect, in some yet unknown ways, the complexities of mitochondrial and cellular physiology. Overall, this work suggests that much remains unknown about the details of mitochondrial DNA replication in different biological states, and the method established here opens up a new avenue in the study of the replication of mitochondrial and potentially other genomes.

## 1. Introduction

A mitochondrion represents a crucial eukaryotic organelle formed by endosymbiosis that retains many of the prokaryotic features of its ancestor [1]. One such hallmark feature is the circular nature of the mitochondrial genome, which is common across the entire animal kingdom. The proper replication of the mitochondrial genome is key to the normal functioning of this organelle, which in turn is key to cell survival [2,3]. Unsurprisingly, a number of diseases have been associated with defects in the replication and maintenance of mitochondrial DNA [4,5,6]. Despite having a relatively small (~16.5 kb in mammals) and simple genome, the debate over which models most faithfully describe mitochondrial DNA replication continues [7,8,9,10,11,12,13,14,15]. However, all existing models are based on the premise that, unlike the replication of mitochondrial DNA in some plants or fungi, which may employ a recombination-dependent mitochondrial replication mechanism [16,17], the replication of the mitochondrial genome in humans and other metazoans depends on the initiation of DNA copying from specific regions within the genome, i.e., the origins of DNA replication. Therefore, the identification and characterization of the origins is a core step in the elucidation of the replication mechanism of mitochondrial genomes.

The best understood metazoan replication origins are the heavy (H) strand origin OH located in the major noncoding region (NCR), and the light (L) strand origin OL located approximately two-thirds across the genome relative to OH [18]. In the most accepted model of the replication of animal mitochondrial DNA—the strand displacement model—the H and L strands are synthesized asynchronously and continuously from OH and OL, respectively [7,8]. Mitochondrial DNA replication is initiated unidirectionally at OH to produce the leading H strand. Meanwhile, the parental H strand, as the lagging strand template, is covered and protected by mitochondrial single-stranded DNA-binding protein (mtSSB) [19,20]. When replication progresses to approximately two-thirds of the mitochondrial genome, the L strand origin OL on the parental H strand forms a stem-loop structure, and L strand replication commences [21,22]. Additional studies have found ribonucleotide incorporation throughout the lagging strand (RITOLS) during mitochondrial DNA replication and suggested the RITOLS/bootlace model, in which RNA instead of mtSSB is used to coat the parental H strand, but replication is still initiated from OH and potentially also OL [14,23]. An alternative synchronous model, called the strand-coupled model, stipulates the simultaneous synthesis of the two strands, where the leading H strand is synthesized continuously while the lagging L strand is synthesized discontinuously [9]. However, the existence of origins of replication is also crucial in this model, which stipulates that the leading strand is initiated either at or near OH, while the lagging strand is initiated from multiple sites [9]. A later study from the same group modified this model and suggested that the initiation is bidirectional and located downstream of OH [10], resulting in a refined model called strand-coupled theta (θ) replication [10]. Besides OH and OL, a third replication origin, Ori-b, located a few hundred nucleotides downstream of OH and was identified later and proposed to be bidirectional [11]. Later studies, however, reported unidirectional initiation from Ori-b [23,24], thus the unidirectional vs. bidirectional nature of Ori-b remains unresolved.

Mitochondrial replication origins have been studied extensively, using a variety of traditional molecular biological and physicochemical techniques, such as primer extension, two-dimensional agarose gel electrophoresis (2D-AGE) [10,11], atomic force microscopy [12,25,26,27], ligation-mediated polymerase chain reaction (LM-PCR) [11,28], restriction endonuclease digestion or 5′ end labeling followed by electrophoresis and fluorography/autoradiography [21,29,30], or in silico prediction [31]. While invaluable in uncovering the key molecular aspects of mitochondrial replication, such methods do not have the sensitivity of more modern techniques based on next-generation sequencing (NGS). To our knowledge, there is only a single study that has profiled initiation sites using a highly sensitive NGS-based technique; however, that work was not focused on the characterization of replication origins [24]. Therefore, in this study, we established and validated a novel approach based on NGS to map sites of the initiation of mitochondrial DNA replication with a nucleotide-level resolution. Using this approach, we identified complex patterns of replication start sites within the three annotated mammalian origins that showed reproducible cell-type specificity under specific biological conditions. These results have several implications for mitochondrial DNA replication and mitochondrial biology in general and show that much still remains unknown about the intricacies of mitochondrial DNA replication in different species. In this respect, the method developed here can be readily applied for the precise characterization of annotated and potentially unannotated origins in different cell types or species that could provide new insights into the intricacies of the replication mechanisms of mitochondrial and, theoretically, other genomes.

## 2. Results

### 2.1. Establishment and Validation of an NGS-Based Strategy for the Detection of Mitochondrial DNA Replication Origins

**Figure 1:**
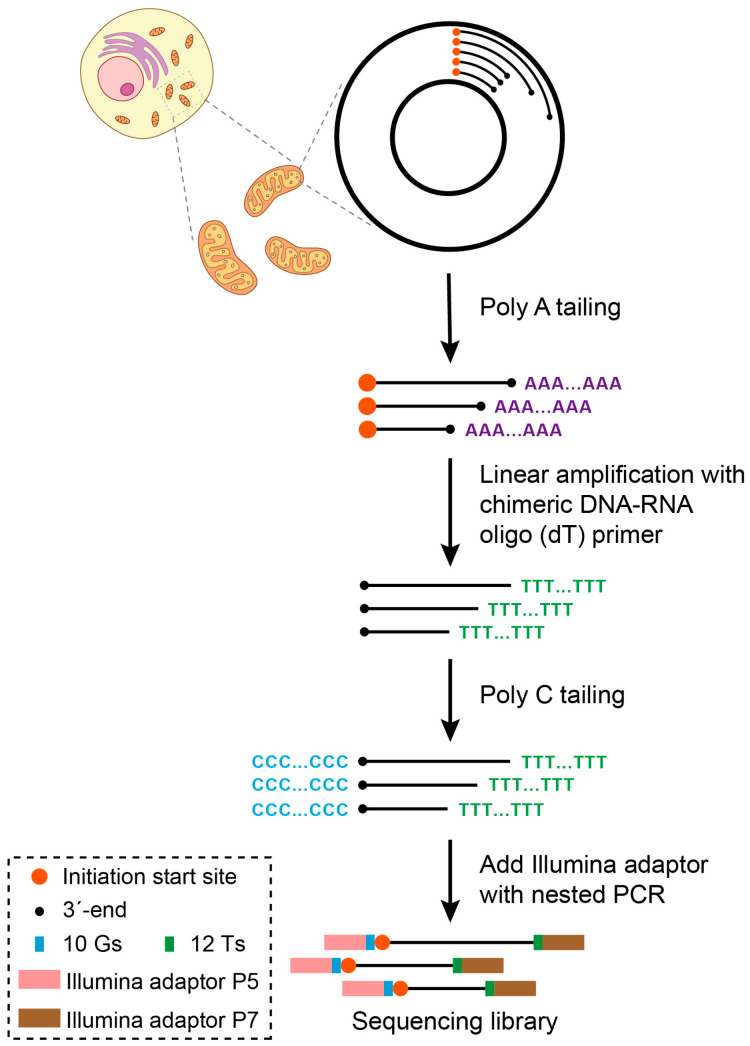
Diagram illustrating the key steps of SSiNGLe-5′ES (SSiNGLe-5′ end sequencing). DNA molecules with 3′-OH ends (black dots) are tailed with polyA by terminal transferase (TdT) and linearly amplified with chimeric DNA-RNA oligo (T) primer. The products of linear amplification are tailed with polyC by TdT. Then, the Illumina adaptors are added with nested PCR to the DNA molecules that have both polyA and polyC tails using oligonucleotides containing 12 thymidines (Ts) and 10 guanosines (Gs). Following NGS, the positions of the replication start sites (red dots) are defined in read pairs that start with both 12 Ts and 10 Gs as coordinates of the bases located immediately downstream of the 10 Gs.

During the process of DNA replication, a certain fraction of nascent DNA is represented by molecules that are in the process of being synthesized, thus representing the intermediates of DNA replication. The intermediates derived from the same initiation site would share a common 5′ end (Figure 1). Therefore, prior to the completion of the replication and resealing of the replicated strands to generate circular genomes, the positions of such free 5′ ends would mark the sites of replication initiation. Thus, a technique that could map the 5′ ends of short, sub-genomic DNA species should be able to identify sites of replication initiation as specific nucleotides in the mitochondrial genome where the signal from the 5′ ends is enriched above the general background. To achieve the detection of the free 5′ ends in mitochondrial DNA, we developed SSiNGLe-5′ES (SSiNGLe-5′ end sequencing) based on the SSiNGLe method, which was previously established by us to detect single-strand breaks (SSBs) with the 3′-OH termini in DNA [32]. Replication intermediates resemble DNA sequences with SSBs by virtue of having 3′-OH termini and thus should be readily detectable by SSiNGLe. However, instead of mapping the 3′-OH termini, the approach used here focused on mapping the 5′ termini (Figure 1). First, the 3′-OH termini representing the ends of mitochondrial replication intermediates were tagged with polyA tails using terminal transferase (TdT, Figure 1). For this purpose, we used purified total cellular DNA as the starting material, as opposed to the crosslinked nuclei used in the original SSiNGLe protocol [32]. Additionally, no fragmentation or other preprocessing steps were performed on the purified DNA prior to tailing. Second, linear amplification with the chimeric DNA-RNA oligo (T) primer was performed to reach the 5′ ends of the DNA, followed by the 3′ end tailing of the amplicons with polyC (Figure 1). Finally, Illumina sequencing adaptors were added with nested PCR followed by NGS and bioinformatics analysis to extract the locations of the 5′ ends of mitochondrial replication intermediates from the aligned reads (Figure 1).

**Figure 2:**
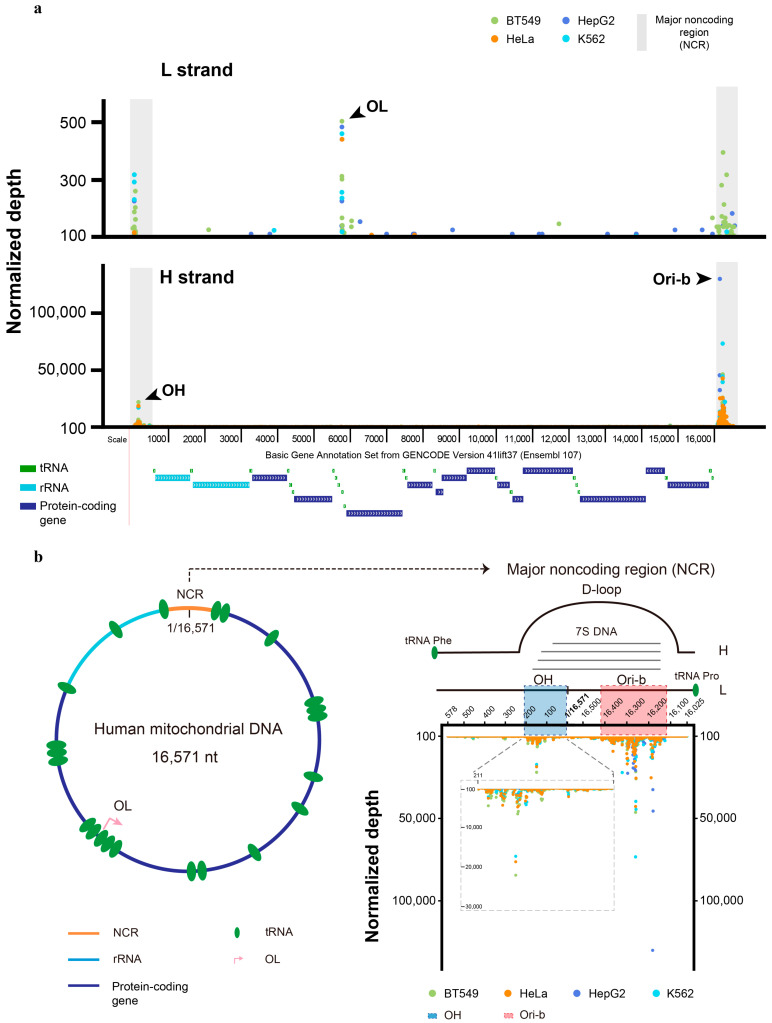
Landscape of mitochondrial 5′ ends in different human cancer cell lines. (**a**) Normalized depths of the SSiNGLe-5′ES signal (Y-axes) derived from the heavy (H) and light (L) strands of the mitochondrial genome in the human cell lines BT549, HeLa, HepG2, and K562. The arrowheads indicate the three annotated origins: OL, OH, and Ori-b. The major noncoding region (NCR) is highlighted in gray, and the genomic annotations are shown below. (**b**) Left, diagram of the mitochondrial genome; right, normalized depth of the SSiNGLe-5′ES signal (Y-axes) derived from the H strand in the NCR region is shown for the indicated cell lines. Positions of OH and Ori-b are highlighted. The inset shows signal in OH using a more sensitive Y-axis scale. Positions of the D-loop and 7S DNA species are also shown. (**a**,**b**) The X-axes represent genomic coordinates. A dot represents the normalized depth of the SSiNGLe-5′ES signal from a single nucleotide position in a particular cell line. Data from the first biological replicate are presented; for the data from another replicate, see Figure S1.

We applied SSiNGLe-5′ES to profile the origins of mitochondrial DNA replication in two biological replicates of each of the four human cancer cell lines: BT549, HeLa, HepG2, and K562. For each base of each strand in the mitochondrial genome, we calculated the normalized density of NGS reads corresponding to the 5′ ends mapped to that position in each replicate in a strand-specific manner. As shown in Figure 2a and Figure S1a, in each replicate of each cell line, the signal was clearly and consistently dominated by the 5′ ends of the H strand intermediates that mapped to the NCR where OH and Ori-b are located. Furthermore, the most prominent 5′ end sites in the NCR formed two distinct clusters that were clearly confined to the annotated boundaries of OH and Ori-b [11] (Figure 2b and Figure S1b).

The highest signal corresponding to the L strand 5′ ends was much lower than that of the H strand 5′ ends. Still, the sites with the most abundant L strand signal could be clearly assigned to the OL and NCR in each cell line (Figure 2a and Figure S1a). It is important to emphasize that our method measures the abundance of the replication intermediates, and since the relative stability of the intermediates produced by different origins is not known, we cannot infer the relative strengths of different origins or the relative fraction of the bidirectional activity of OH and Ori-b (see Section 3). In fact, OH can generate very stable discrete H-strand replication intermediates represented by the short 7S DNA species [33], which likely explains the very strong signal observed in this origin using our method. Since the strength of the Ori-b signal detected by SSiNGLe-5′ES was similar to that of OH (Figure 2 and Figure S1), it appears that such H-strand intermediates are also generated by Ori-b, but not by OL, and not by the L-strand initiation activity of OH and Ori-b, judging by the low signal in OL and on the L strand of OH and Ori-b (Figure 2a, Figures S1a and S2). Still, our results are consistent with at least one previous study that failed to observe the bidirectional nature of Ori-b in certain cell types, including HeLa, the only cell type in common between the two studies [24]. Furthermore, similar to our findings, that report also provided supporting evidence for the bidirectional activity of OH [24].

Taken together, these results strongly argue that SSiNGLe-5′ES could specifically identify the origins of mitochondrial DNA replication, as evidenced by the fact that the five regions with the highest signal corresponding to the initiation sites detected using this method—two for H and three for the L strand—colocalized with the three known origins and were consistent with previous reports of the bidirectional activity for two of them. Therefore, as the next step, we characterized the precision of the detection of the initiation sites.

### 2.2. Nucleotide-Level Analysis of the Initiation Sites within the Annotated Human Origins

**Figure 3:**
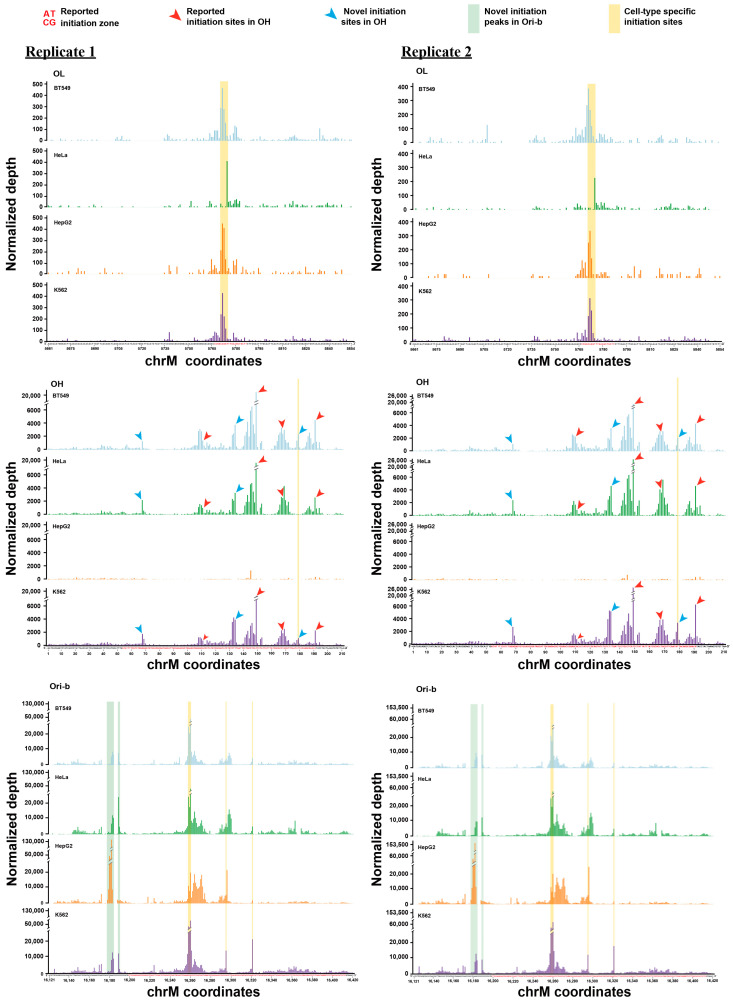
Detailed views of the 5′ end signals in the human mitochondrial replication origins OL, OH, and Ori-b. Normalized depths of the SSiNGLe-5′ES signal (Y-axis) derived from the L (OL) or H (OH and Ori-b) strands of the mitochondrial genome in the human cell lines BT549, HeLa, HepG2, and K562 are shown. The OH and Ori-b regions shown here correspond to the regions highlighted in the right part of Figure 2b. Red letters indicate the reported initiation zones. Reported major initiation sites in OH and those found only in this work are marked by the red and blue arrowheads, respectively. Novel initiation peaks in Ori-b are highlighted in green. Cell-type-specific initiation sites are highlighted in yellow. The X-axes represent genomic coordinates. Data from two biological replicates are presented.

Among the three known human origins, OL has the narrowest annotated initiation zone—several previously found initiation sites have been mapped to a 21-nt region between nucleotides 5767 and 5787, downstream from a conserved stem-loop structure (nucleotides 5731–5764) [28,34]. As shown in Figure 3, the positions where strong SSiNGLe-5′ES signals were detected corresponded very well with the previously annotated initiation zone and were mapped between nucleotides 5764 and 5787, with the 5771–5774 region having the predominant signal (Figure 3).

The sites of replication initiation within OH have been previously mapped to the nucleotides 111, 149, 168, and 191, with positions 149 or 191 reported as the major sites in different studies [11,24,28,29,35]. However, somewhat similar to OL, instead of observing a signal at these discrete positions, we found peaks of multiple adjacent initiation sites spread across OH, similar to the initiation peak found in OL (Figure 3). Still, the positions of the peaks matched very well with those of all the reported initiation sites (Figure 3). Furthermore, consistent with at least one previous report [24], the peak surrounding position 149 had the highest signal in all three cell lines (BT549, HeLa, and K562) with detectable OH signals (Figure 3). Ori-b is a relatively less-studied origin compared to OL and OH, and it was reported to cover a broad region downstream of OH—nucleotides 16,199 to 16,413 [11]. In this region, we could observe several peaks of initiation, with the most prominent ones located at positions 16,178–16,184, 16,189–16,190, 16,257–16,274, and 16,289–16,300 (Figure 3).

In summary, at the nucleotide-level resolution, our results matched quite well with those reported previously for OL and OH, suggesting that the method could faithfully uncover replication initiation sites. Furthermore, as shown in Figure 3, the SSiNGLe-5′ES profiles of the three annotated origins were highly similar between the two biological replicates of each cell line, strongly suggesting the highly reproducible nature of the patterns of replication initiation sites and the method itself. Furthermore, we also found two striking features in these patterns.

First, in addition to the annotated replication sites, we could detect novel initiation peaks. In OH, such sites were represented by peaks surrounding positions 68, 134, and 179 (Figure 3). In Ori-b, we could also observe novel peaks in a nearby region 16,124–16,198, in which two major peaks located at positions 16,178–16,184 and 16,189–16,190 could be detected. These results suggested that the Ori-b origin should be defined as a broader region from position 16,124 to 16,420 (Figure 3).

**Figure 4:**
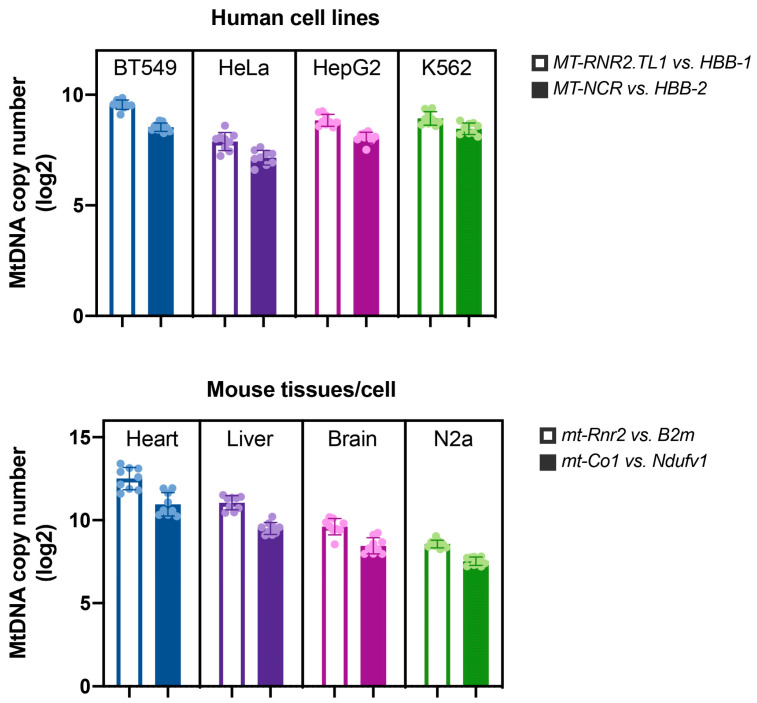
Mitochondrial DNA copy number in different samples. The Y-axes represent mitochondrial DNA copy numbers in the indicated human and mouse cell lines or tissues on the log_2_ scale. For each sample, the copy number was measured by two qPCR assays, each using one indicated primer pair designed against the mitochondrial genome and one against the nuclear genome (Supplementary Table S1, Section 4). Error bars indicate SD of 9 measurements (shown by dots): 3 technical replicates for each of the 3 biological replicates.

Second, we observed cell-type specificity of the initiation patterns in all three origins at both global and local levels. The former was represented by the differences in the whole origin level, as exemplified by the virtual absence of replication initiation peaks within the OH region in HepG2, as shown in detail in Figure 3. Importantly, the absence of the SSiNGLe-5′ES signal in OH was unlikely to be caused by technical issues since the magnitude of the signal in the other two origins, OL and Ori-b, in HepG2 was similar to that in the other cell lines (Figure 3). Interestingly, the L strand signal in the NCR was confined to the annotated boundaries of OH and Ori-b [11] (Figure 2a and Figure S1a), suggesting that the latter is indeed a bidirectional origin, as reported in at least one previous study [11]. However, a strong L strand signal in Ori-b was found only in one cell line (BT549), suggesting that the bidirectional nature of this origin may be cell-line-specific (Figure S2a). In contrast, a strong L strand signal in OH could be observed in three out of the four cell lines—BT549, HepG2, and K562 (Figure S2b). Furthermore, the copy number of mitochondrial DNA in HepG2 detected by quantitative real-time PCR (qPCR) was comparable to that in the other three cell lines (Figure 4). These observations argued against technical reasons for the absence of the signal at OH and suggested that either this origin is not active in HepG2 or it does not generate stable replication intermediates.

Local-level differences among the cell lines were represented by differences in either nucleotide-level patterns or the existence of specific peaks. For example, the major initiation sites in OL could be consistently detected at nucleotides 5771–5773 in HepG2, BT549, and K562; however, HeLa contained only a major initiation site at position 5775 (Figure 3). In the case of OH, the novel peak at position 179 was consistently found in BT549 and K562 but was absent in HeLa (Figure 3). All major initiation peaks at Ori-b exhibited cell-line specificity. For example, the initiation peak at positions 16,256–16,274 exhibited preferential initiation at the nucleotides 16,258–16,260 in BT549, K562, and HeLa, while exhibiting a relatively even initiation pattern across the entire peak in HepG2 (Figure 3). The initiation sites 16,295 and 16,321 also showed a cell-type-specific pattern, with the former being more dominant in HepG2 and K562, and the latter being more dominant in K562 (Figure 3). Additionally, HepG2 had a much stronger initiation signal in the novel Ori-b peaks located at positions 16,178–16,184 and 16,189–16,190 compared to the other three cell lines (Figure 3).

Overall, these results have shown that replication initiation patterns could be specific to different human cancer cell lines. For the next step, we extended these studies to three normal mouse tissues to investigate whether the cell-type specificity of mitochondrial initiation sites could be detected in different species and in normal, nonmalignant tissues.

### 2.3. Dynamic Pattern of the Initiation Sites Is a Common Feature of Mammalian Mitochondria

**Figure 5:**
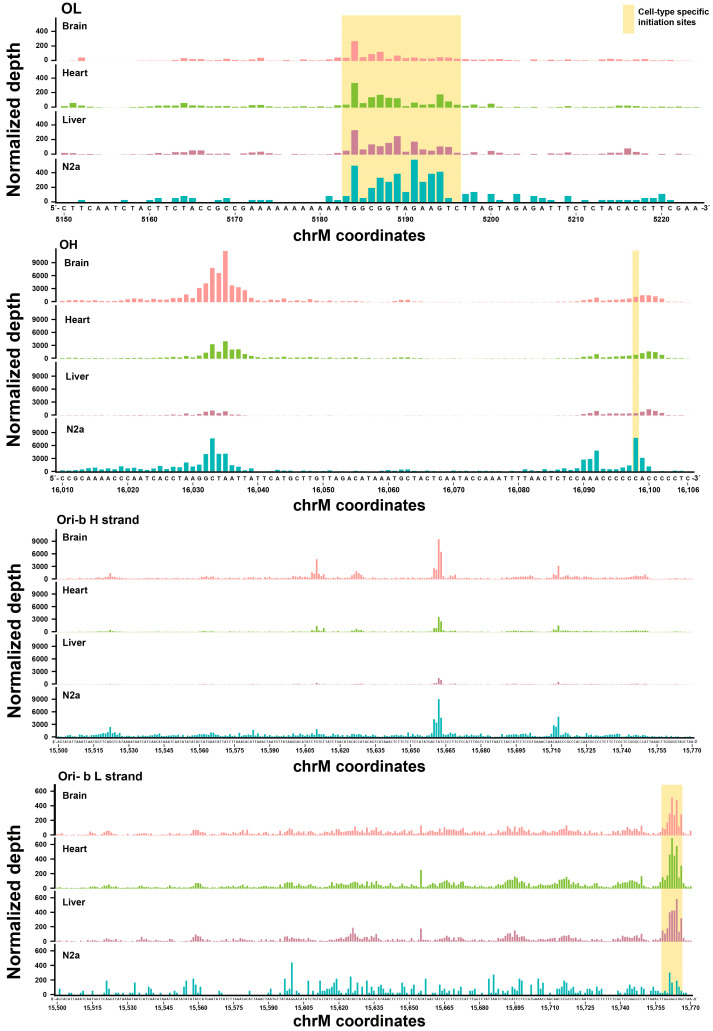
Detailed views of the 5′ end signals in the mouse mitochondrial replication origins OL, OH, and Ori-b. Normalized depths of the SSiNGLe-5′ES signal (Y-axes) derived from the L (OL and Ori-b) or H (OH and Ori-b) strands of the mitochondrial genome in the mouse tissues and the N2a cell line are shown. Cell-type-specific initiation sites are highlighted in yellow. The X-axes represent genomic coordinates. Data from the first biological replicate are presented; for the data from the other two replicates, see Figure S3.

For the next step, we applied SSiNGLe-5′ES to profile the initiation sites in mouse brain, heart, and liver tissues from three different 7-week-old animals. We also included the neuroblastoma cell line N2a as a representative mouse cancer cell line. Consistent with previous reports in mice [23,36,37] and also with the human data described above, we could observe strong initiation signals on the H strand represented by two clusters of peaks corresponding to the OH and Ori-b regions in the NCR (Figure 5, Figures S3 and S4), and on the L strand in the OL annotated region (Figure 5 and Figure S3) in all mouse samples. Since, to our knowledge, the mouse replication initiation sites have not been as comprehensively studied as the human ones, we could not compare our mouse results with the previously published data at a fine level as we have done for the human origins. Nonetheless, the availability of the mouse data has allowed us to draw the following conclusions.

First, similar to the human results, we have also observed broad replication zones in all three origins, patterns of which were highly consistent among the different biological replicates of the same sample type (Figure 5 and Figure S3). The same as in humans, the OL replication zone was represented by one peak of adjacent initiation sites, while the OH and Ori-b zones were represented by multiple such peaks. Second, similar to the human results, we found consistent local and global differences in the usage of the origins among different biological samples. For example, at the global level, we have observed that the Ori-b signal is obviously higher in the brain and N2a compared to the heart and especially the liver (Figure 5 and Figure S3). Furthermore, liver had the lowest overall signal in OH among the four sample types (Figure 5 and Figure S3). Interestingly, these differences could not be explained by a lower amount of mitochondrial DNA present in the liver and heart since qPCR analysis found that these tissues had similar mitochondrial DNA copy numbers, which were actually slightly higher than those in the brain and N2a (Figure 4, see Section 3).

**Figure 6:**
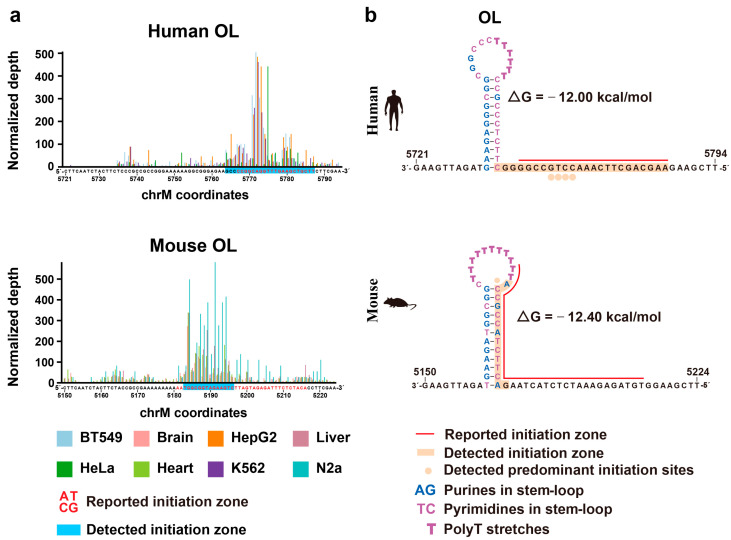
Comparison of OL in humans and mice. (**a**) The normalized depths of the SSiNGLe-5′ES signal (Y-axis) from the L strand of the human and mouse mitochondrial genomes are shown. Red letters indicate the reported OL initiation zones. Major OL initiation zones detected in this study are highlighted in blue. The X-axes represent genomic coordinates. Data from the first biological replicate are presented; for the data from the other replicates, see Figure S5. (**b**) Stem-loop structures of OL in humans and mice are shown. The red lines indicate the reported OL initiation zones. The initiation zones detected in this work are highlighted in orange; the orange dots indicate the predominant initiation sites in the human OL detected in this work. Within the stem–loop regions, purines and pyrimidines are marked, respectively, in blue and purple. Bold letters indicate the polyT stretches.

We could also observe differences at the local level in all three origins, as illustrated by OH (Figure 5 and Figure S3). In mice, OH has two major discernible OH initiation peaks at positions 16,031–16,037 and 16,090–16,102 (Figure 5 and Figure S3). The signal from the former initiation peak was significantly higher than that from the latter in the brain, while the differences between the two peaks were much less pronounced in the heart, liver, and N2a (Figure 5 and Figure S3). Interestingly, we also observed reproducible nucleotide-level differences in the patterns of the two initiation peaks between the N2a cell line and the three tissues (Figure 5 and Figure S3). For example, in OH, the strongest signal in the N2a cell line occurred at position 16,098, which was not the case in all three normal tissues (Figure 5 and Figure S3). We could also observe nucleotide-level differences in the OL. Although the initiation zone (5183–5196) detected in N2a was the same as in other mouse tissues, the shape of the peak was notably different: while the tissues (especially the brain) preferred initiation at position 5184, the initiation profile in N2a was more even across the entire zone (Figure 5, Figure 6a and Figure S3). The shape of the peak was also different among the normal mouse tissues: while the shape was similar in heart and liver, it was clearly different in the brain (Figure 5 and Figure S3). The N2a cell line also had another difference in the L strand pattern: we found a consistent peak (15,758–15,766) on the L strand in the Ori-b region with a similar position and relative signal in all three tissues, which was, however, absent in the N2a cell line (Figure 5, Figures S3 and S4).

Third, we found differences between the two species in the initiation profiles of OH and OL. While human OH contained multiple initiation peaks (Figure 3), its mouse counterpart contained only two major peaks (Figure 5 and Figure S3). However, the absence of conservation in the NCR of the two species precluded us from conducting a detailed nucleotide-level comparison of the locations of the OH and Ori-b peaks. We also detected some interesting differences between humans and mice with regard to the patterns of the initiation sites in OL. OL forms a stem-loop structure on the template strand that is critical for its function [22,34]. Based on the current model, the polyT stretch within the loop of the human OL (Figure 6) acts as a transcription start region for the short primer RNA species made by the mitochondrial primase RNA polymerase POLRMT. The latter is replaced by DNA polymerase within a region immediately downstream from the stem, the replication initiation zone [28,34], where we have also observed a strong 5′ end signal in the human samples, consistent with previous studies (Figure 3 and Figure 6). On the other hand, the initiation zone in the murine OL started immediately downstream of the polyT stretch in the loop and spanned the stem-loop region, with the predominant initiation site (position 5184) located at the junction of the loop and stem for the three mouse tissues (Figure 5 and Figure 6). In the N2a cell line, however, strong signals could be observed across the whole initiation zone (Figure 5 and Figure 6). Interestingly, a 1979 study [21], using lower-resolution techniques available at that time, also found the murine OL initiation signal in the stem-loop region, suggesting potential differences in the initiation mechanisms between the two species (see Section 3). It is worth mentioning that in that study, the initiation zone was detected in a wider region spanning both the stem-loop and downstream regions (chrM: 5181-6-5216) [21], while in our study, the downstream region has a much weaker signal than the stem-loop region.

## 3. Discussion

In this study, we developed an NGS-based method, SSiNGLe-5′ES, that could be used to precisely map the initiation sites of DNA replication and thus investigate the properties of annotated and unannotated origins of replication at nucleotide-level resolution. We validated the procedure by virtue of applying it to the human mitochondrial genome, which has the best studied origins of replication and for which the positions of some of the major initiation sites have been previously mapped. Indeed, we have found that the genomic locations corresponding to the most prominent signals obtained by the method matched very well with the locations of the three annotated origins of replication, and that there was precise correspondence at the nucleotide level between the initiation sites found in this study and those reported previously. Similar results were also obtained for the mouse mitochondrial genome. These results strongly suggest that SSiNGLe-5′ES can indeed accurately detect sites of initiation of the replication of the mitochondrial genomes of different species.

Interestingly, the method could provide novel insights even for well-annotated origins. First, we found that replication initiation sites within OH and Ori-b are represented by the peaks formed by adjacent initiation sites rather than by discrete sites. In this regard, each peak appears to represent an initiation area similar to the initiation zone of OL. Therefore, OH and Ori-b could be regarded as multi-initiation peak origins, while OL could be a single initiation peak origin. Second, we found novel initiation sites within and immediately adjacent to human OH and Ori-b, respectively. Third, in terms of the ongoing debate regarding the bidirectional nature of Ori-b [11,23,24], our results suggest that it can indeed be bidirectional in some but not all human cell lines. Fourth, and related to the previous point, our method identified cell-type specificity in the usage of specific initiation peaks and sites within the same origin and/or in the total relative amount of the initiation signal in different origins. For example, we found no signal in OH in the HepG2 cell line, while the signal from Ori-b in that cell line was quite strong. Furthermore, one initiation peak in that origin was present in BT549 and K562 but not HeLa. It is important to emphasize that differences among the various cell types also occur on a very fine nucleotide-level scale. One of the best illustrations of this phenomenon is a very subtle yet consistent difference in the initiation site pattern in the human OL. While three human cell lines (HepG2, BT549, and K562) consistently exhibit a maximum signal at 5771–5773, the signal in these positions in HeLa was not detectable. In contrast, that cell line contained only one major initiation site at position 5775.

These results bring about natural questions: what is (are) the underlying molecular reason (s) behind the differences in the patterns of the initiation sites, and what is the biological meaning of these differences? The answers to both of these questions are currently unknown. Our results suggest that the global differences in the origin-wide SSiNGLe-5′ES signal may not be directly connected to the steady-state amount of mitochondrial DNA per cell, as evidenced by the mouse liver data. In that tissue, the cumulative strength of the SSiNGLe-5′ES signal was significantly lower in both OH and Ori-b compared to the mouse heart, brain, and N2A cell line; however, the copy number of mitochondrial DNA in the liver was not lower than that in the latter two sample types and was similar to that in the heart.

In this context, it is worth re-emphasizing that our method measures the abundance of relatively short replication intermediates. Stable replication intermediates such as 7S DNA species would dominate the SSiNGLe-5′ES signal; however, their abundance would depend not only on the activity of the corresponding origin, but also on the rate of termination of DNA synthesis and the stability of the intermediates in the cell. Thus, the relationship between the abundance of such intermediates and the activity of the corresponding origins might be complex. Therefore, the magnitude of the SSiNGLe-5′ES signal cannot be easily correlated with the activity of the corresponding origin. Finally, it is currently hard to predict the biological consequences of this subtle shift in the locations of the initiation sites.

However, despite all these caveats, it is also important to highlight the consistency of the patterns in different biological replicates of the same cell type, which at the very least suggests the existence of some differences in the mitochondrial replication machinery and/or mitochondrial, and even cellular, physiology that are reflected in the differences in the patterns of the initiation sites. As shown by the mouse tissue, these differences cannot be easily revealed by measuring the copy number of mitochondrial genomes, nor can all these differences be revealed by methods that lack nucleotide-level precision.

Mitochondria play key roles in multiple aspects of cancerogenesis [38]. In this respect, it is interesting that we observed major differences among the SSiNGLe-5′ES profiles of the human cancer cell lines and also strong differences between the profiles of the mouse N2a neuroblastoma cell lines and the mouse normal tissues. One possible explanation for these differences could be the effect of mutations that have been often found in mitochondrial DNAs in cancers [39]. It is also possible that mutations in the nuclear-encoded components of mitochondrial replication machinery could be at least in part responsible for the observed differences in the replication patterns. Mutations in the mitochondrial or nuclear genomes could be either somatic or germline [39]. In this regard, it would be very interesting to conduct a study where the SSiNGLe-5′ES profiles would be correlated with the presence of various mutations in the mitochondrial or nuclear genomes across multiple cancer cell lines and tissues. However, it is also important to mention that mutations cannot be the only reason for the differences in the mitochondrial replication profiles since we could observe these differences among the different tissues obtained from the inbred mice. Thus, other potential factors, such as the expression levels of the various components of the mitochondrial replication machinery or their isoforms, could result in such differences. In summary, while the molecular mechanisms and biological significance of the differences in the patterns of the initiation sites are not known, their existence should highlight the currently under-appreciated field of the dependence of mitochondrial DNA replication on cell type and other biological factors.

Fifth, we identified differences in the patterns of initiation sites between different species. For example, in agreement with an earlier study [21], we found that the initiation peaks differed significantly between the human and mouse OL. These observations resonate with those from a previous study in which an in vitro system that reconstituted human mitochondrial replication failed to initiate replication from the mouse OL, while it could initiate replication from both human and bovine OLs [30]. In this respect, while the mice are more phylogenetically close to humans than cows, the sequence around mitochondrial OL in mice is more divergent from humans than the bovine homolog, both regarding the loop sequence (the site of initiation by the mitochondrial primase) and the sequence immediately downstream of the stem-loop structure, which may serve as the recognition site for RNase H and be critical for transition from RNA to DNA synthesis [30,34]. Taken together with these observations, our results suggest that the molecular mechanisms of initiation at the mouse OL may be different from those operating at the human OL.

## 4. Materials and Methods

### 4.1. Experimental Design

To identify the initiation sites of mitochondrial DNA replication derived from known and unannotated replication initiation sites, we established an NGS-based method, SSiNGLe-5′ES, based on the SSiNGLe-ILM protocol from our previous study [32], that can detect free 5′ ends of mitochondrial replication intermediates with a high resolution and in a high-throughput manner. The method was applied to multiple human cell lines as well as mice cell line and tissues in order to uncover the fine patterns of initiation sites and explore potential cell-type/tissue specificity of replication initiation events.

### 4.2. Biological Material and DNA Isolation

Human breast cancer cell line BT549 (obtained from National Collection of Authenticated Cell Cultures, Wuhan, China), human cervical carcinoma cell line HeLa (obtained from National Infrastructure of Cell Line Resource, Beijing, China), human liver cancer cell line HepG2 (obtained from Cell Bank of Chinese Academy of Sciences, Shanghai, China), and human chronic myeloid leukemia cell line K562 (obtained from Cell Bank of Chinese Academy of Sciences) were maintained in RPMI 1640 (Gibco, Billings, MT, USA) supplemented with 10% heat-inactivated fetal bovine serum (ExCell Bio, Montevideo, Uruguay) and 1% penicillin–streptomycin (Gibco). Mouse neuroblastoma cell line N2a (obtained from National Infrastructure of Cell Line Resource) was cultured in MEM/EBSS (HyClone, Logan, UT, USA) supplemented with 10% heat-inactivated fetal bovine serum (ExCell Bio) and 1% penicillin–streptomycin (Gibco). All cell lines were cultured at 37 °C in 5% CO_2_. Adult male C57BL/6J mice (*Mus musculus*) were used to extract brain, heart, and liver tissues, which were then frozen in liquid nitrogen. The mouse tissues were cut into pieces and ground with mortar in liquid nitrogen. The ground tissues were stored at −80 °C prior to DNA isolation. The animal experiments were approved by the laboratory animal management ethical review board of the School of Medicine, Huaqiao University.

DNA was isolated using a TIANamp Genomic DNA Kit (Tiangen, Beijing, China) with RNase A (Tiangen) treatment from either 2 million cells (cell lines) or 30–60 mg of ground powder (tissues). In this work, we used two and three biological replicates for the human and mouse samples, respectively. The concentrations of DNA were measured using Qubit 3.0 fluorometer using an Equalbit dsDNA HS Assay Kit (Vazyme, Nanjing, China).

### 4.3. SSiNGLe-5′ES: Wet Lab

The biochemical part of SSiNGLe-5′ES was based on the SSiNGLe-ILM protocol from our previous study [32]. Briefly, 100 ng of the purified DNA was denatured at 95 °C for 5 min in 19 µL volume containing 2 µL of 10× TdT buffer and 2 µL of 2.5 mM CoCl_2_, followed by rapid snap-cooling on ice. TdT (5 units, NEB) and 2 µL of 10 mM dATP (Takara, Kusatsu, Japan) were then added to denatured DNA to a total volume of 22 µL and incubated at 37 °C for 30 min. To block free 3′-OH ends, the sample was mixed with 2 µL of 10 mM of each ddNTP (Roche, Basel, Switzerland) and incubated at 37 °C for an additional 30 min, followed by enzyme inactivation at 70 °C for 10 min. The tailing mix was then purified with 2× volumes of VAHTS DNA Clean Beads (Vazyme) to a final volume of 15 μL and mixed with 2 µL of 10× Taq buffer (Tiangen), 1.6 µL of 2.5 mM dNTP mix (Takara), 1 µL of 10 µM of polyT primer (a chimeric DNA-RNA oligo-d(T)50-r(T)3 oligonucleotide), and 1 unit of Taq DNA polymerase (Tiangen). The amplification conditions were as follows: initial denaturation at 94 °C for 3 min, followed by 10 cycles of denaturation at 94 °C for 30 s, annealing at 55 °C for 30 s, and extension at 72 °C for 30 s. The PCR products were purified with 2× volumes of VAHTS DNA Clean Beads (Vazyme) and tailed with dCTP (Takara) with the same procedure used for polyA tailing, followed by blocking of 3′-OH ends as described above. The tailing products were purified with 2× volume of VAHTS DNA Clean Beads (Vazyme) and all purified DNA was PCR-amplified with P5_10N10G (CTACACGACGCTCTTCCGATCTAGTTGCGGATGGGGGGGGGGHN) and P7_T12 (GTTCAGACGTGTGCTCTTCCGATCTTTTTTTTTTTTTVN) primers with the following amplification conditions: initial denaturation at 94 °C for 3 min; 1 cycle of denaturation at 94 °C for 30 s, annealing at 55 °C for 1 min, and extension at 72 °C for 1 min; 1 cycle of denaturation at 94 °C for 30 s, annealing at 37 °C for 1 min, and slow ramp at 2 °C per minute to 72 °C; 30 cycles at 94 °C for 30 s, annealing at 60 °C for 30 s, and extension at 72 °C for 30 s; and final extension at 72 °C for 7 min. The amplified DNA was purified with 2× volumes of VAHTS DNA Clean Beads (Vazyme) to a final volume of 17 μL of water, and 4 µL PCR products were used as the template for the second round of nested PCR with lIlumina_P5 (5′-AATGATACGGCGACCACCGAGATCTACACTCTTTCCCTACACGACGCTCTTCCGATCT-3′) and primer Illumina_P7 (CAAGCAGAAGACGGCATACGAGAT (bar_code) GTGACTGGAGTTCAGACGTGTGCTCTTCCGATCT) as primers. The PCR amplification was conducted under the following conditions: 94 °C for 3 min; 6 cycles of 94 °C for 30 s, 55 °C for 30 s, and 72 °C for 30 s; and 72 °C for 7 min. The PCR products were purified with 2× volumes of VAHTS DNA Clean Beads (Vazyme) and used for NGS on Illumina platforms NovaSeq 6000 (Novogene, Beijing, China) using paired-end 150 bp (PE150) strategy and outsourced to on a 5-gigabase (GB) scale.

### 4.4. SSiNGLe-5′ES: Bioinformatics

Only paired-end raw reads, where read 1 started with 9–11 Gs, read 2 started with 11–13 Ts, and each base of each read with Phred quality score >20 were selected. These reads were then aligned to the mitochondrial genome (chrM) sequences of GRCh37/hg19 and GRCm38/mm10 assemblies of the, respectively, human and mouse genomes downloaded from UCSC Genome Browser [40], respectively, using BWA-MEM (v0.7.12) with the default settings. Only pairs of reads where both reads 1 and 2 uniquely mapped to the genome with an appropriate configuration and spacing were kept. The 5′ end was defined as the first base after 9–11 Gs in read 1. The read count of each position was obtained and normalized depth was calculated, with the read count of each position being normalized to the total number of reads aligned to the mitochondrial genome in that sample.

### 4.5. In Silico OL Structure Analysis

The in-silico structure analysis of human and mouse OL was performed using the RNAfold Webserver of the Vienna RNA Websuite [41,42]. The calculation of △G (minimum free energy) was performed on the same webserver using the DNA parameters under the default settings [41,42].

### 4.6. Mitochondrial DNA Copy Number Measurements

Measurements of the copy number of mitochondrial DNA were performed using previously published qPCR techniques [43,44,45,46] on the same DNA preparations used for the SSiNGLe-5′ES analyses. Briefly, each sample was assayed by qPCR with four separate primer pairs (two for nuclear and two for mitochondrial genome of the corresponding species, Supplementary Table S1) using PowerUp SYBR Green Master Mix (Life Technologies, Carlsbad, CA, USA) on a Mx3005P cycler (Agilent Technologies, Santa Clara, CA, USA). For each sample type and primer pair, assays were performed on three biological replicates, each represented by three technical replicates. For each qPCR reaction, 500 pg of genomic DNA was used as the template. The copy number of mitochondrial DNA was calculated as 2 × 2^[−(Ct_mito_ − Ct_nuc_)], where Ct_mito_ and Ct_nuc_ indicate the Ct values obtained from primer pairs targeting the mitochondrial and nuclear sequences, respectively [43,44,45,46,47] (Supplementary Table S1).

## 5. Conclusions

In summary, the discovery of novel initiation sites within the known origins in the mitochondrial genomes of the two species suggests that additional studies using SSiNGLe-5′ES and similar methods are required to fully map and understand the mechanisms of the replication of mitochondria in different species. Furthermore, our observations suggest that the patterns of initiation sites can be an additional source of molecular biomarkers that are specific to a particular biological state. Therefore, the profiling activity of different origins using SSiNGLe-5′ES in multiple sample types could provide further insights into the intricacies of mitochondrial DNA replication and add a new dimension to the understanding of the biological complexities of the different biological systems that cannot be revealed by other approaches, for example, those that measure the mitochondrial DNA copy number. The method is relatively simple, has high throughput, and can be easily scaled to a large number of samples. While SSiNGLe-5′ES was only applied to human and mouse samples in this work, it can be used to characterize mitochondrial initiation sites in other species. Conceivably, it can be also applied to the genomes of other organelles, such as chloroplasts, and even the genomes of other organisms that have major origins of replication, such as bacteria.

## Data Availability

The NGS data were submitted to GEO with accession number GSE220621.

## Supplementary Materials

The following supporting information can be downloaded at: https://www.mdpi.com/article/10.3390/ijms24119711/s1.
