# Supplementary material for: Dynamic Patterns of Mammalian Mitochondrial DNA Replication Uncovered Using SSiNGLe-5′ES
Source: Int J Mol Sci. 2023 Jun 3;24(11):9711. doi: 10.3390/ijms24119711 (PMC10253933; doi:10.3390/ijms24119711)
Supplement: Supplementary file 1 [file ijms-24-09711-s001.zip › ijms-2377051-supplementary_Figures.pdf]

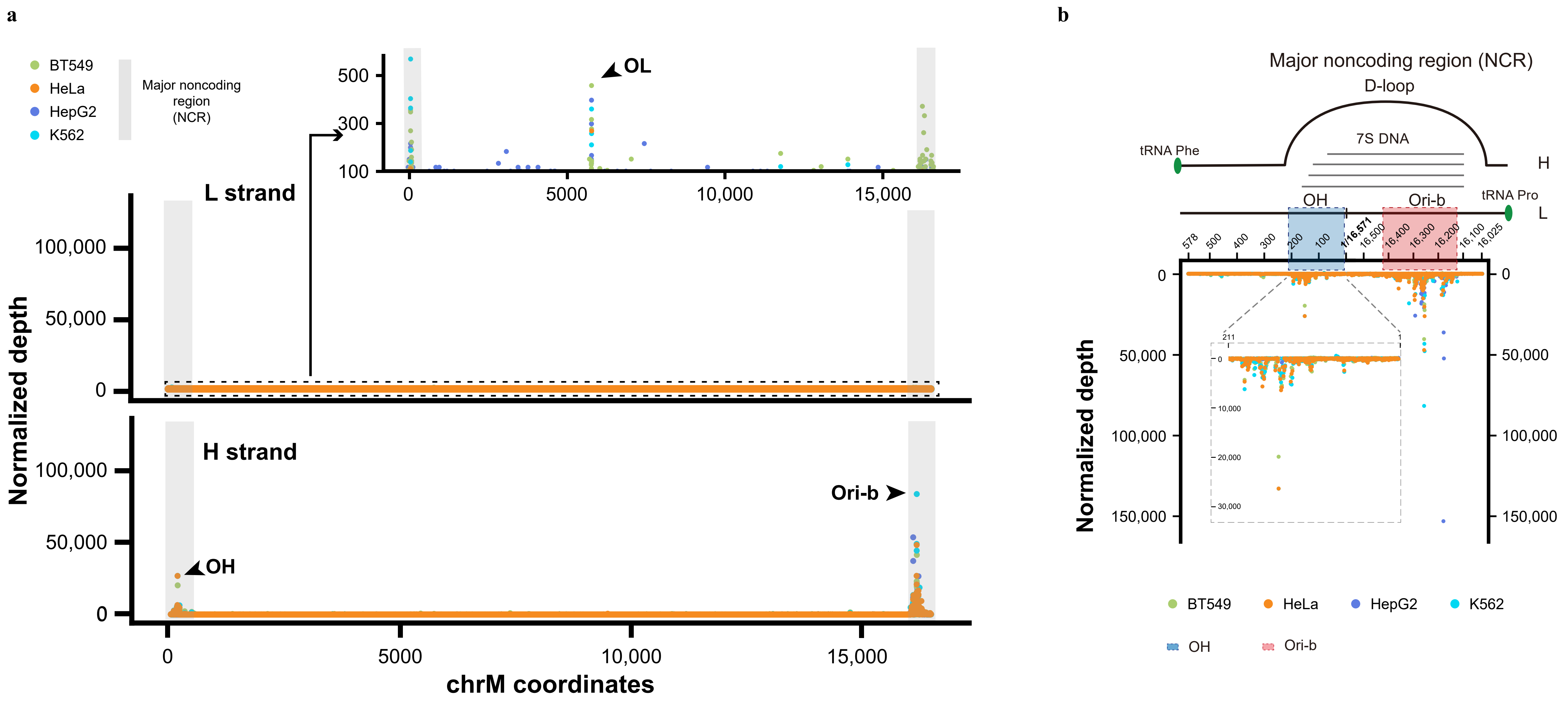

Fig.S1. Landscape of mitochondrial 5'-ends in different human cancer cell lines — the second biological replicate. (a) Normalized depths of the SSiNGLe-5'ES signal (Y-axes) derived from the H and L strands of the mitochondrial genome in the human cell lines BT549, HeLa, HepG2, and K562. The inset shows signal in L strand using a more sensitive Y-axis scale. The arrowheads indicate the three annotated origins OL, OH, and Ori-b. The major noncoding region (NCR) is highlighted in grey. **(b)** Normalized depth of the SSiNGLe-5'ES signal (Y-axis) derived from the H strand in the NCR region is shown for the indicated cell lines. Positions of OH and Ori-b are highlighted. The inset shows signal in OH using a more sensitive Y-axis scale. Positions of the D-loop and 7S DNA species are also shown. **(a, b)** The X-axes represent genomic coordinates. A dot represents the normalized depth of the SSiNGLe-5'ES signal from a single nucleotide position in a particular cell line. The corresponding results from the first biological replicate are shown in Fig.2.

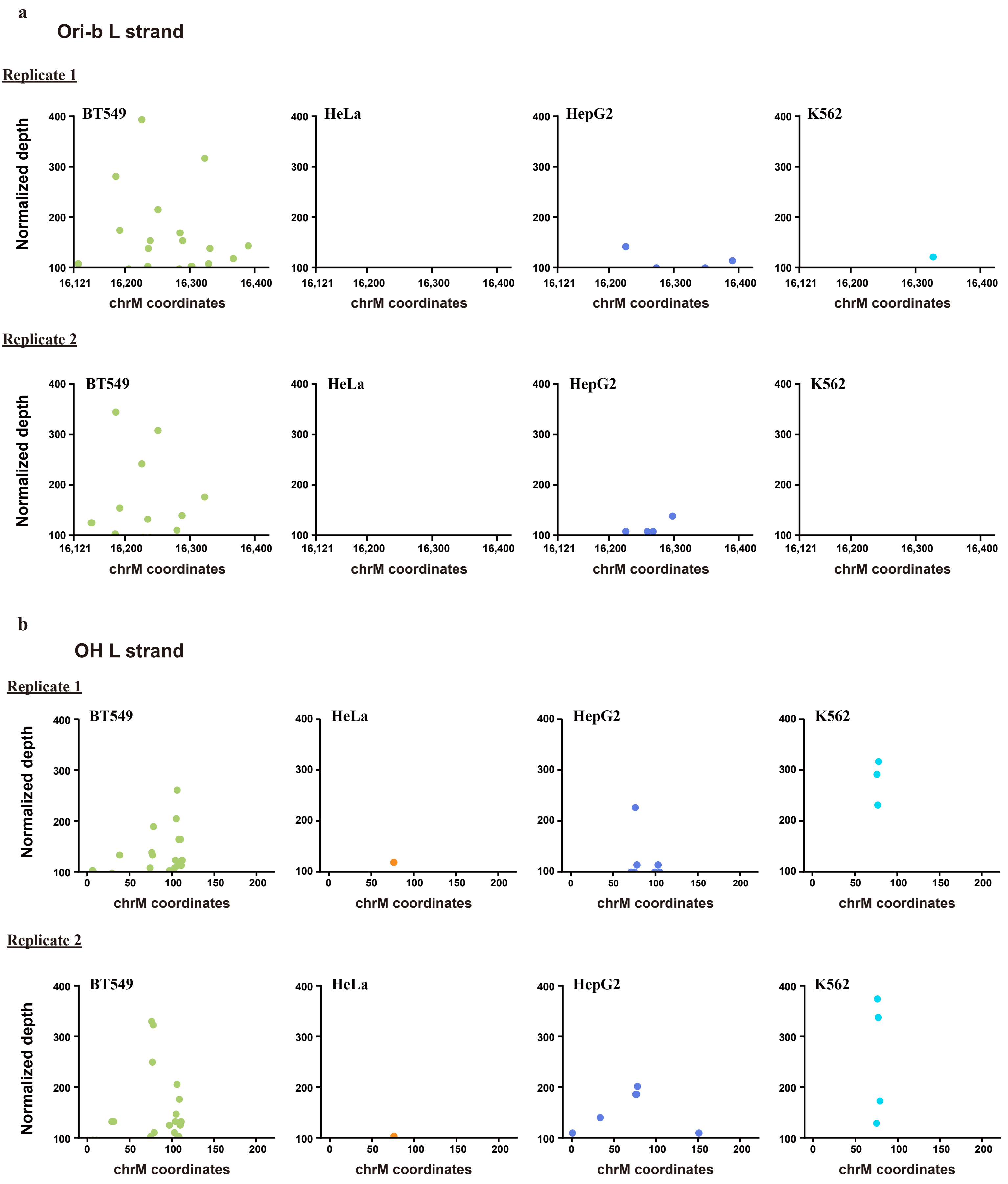

Fig.S2. Detailed views of the L-strand 5'-end signals in the human mitochondrial H-strand replication origins OH and Ori-b in different cancer cell lines. Normalized depths of the SSiNGLe-5'ES signal (Y-axes) derived from the L strand of the mitochondrial genome in the human cell lines BT549, HeLa, HepG2, and K562 in Ori-b **(a)** and OH **(b)** regions. The X-axes represent genomic coordinates. A dot represents the normalized depth of the SSiNGLe-5'ES signal from a single nucleotide position in a particular cell line. Data from two biological replicates are presented.

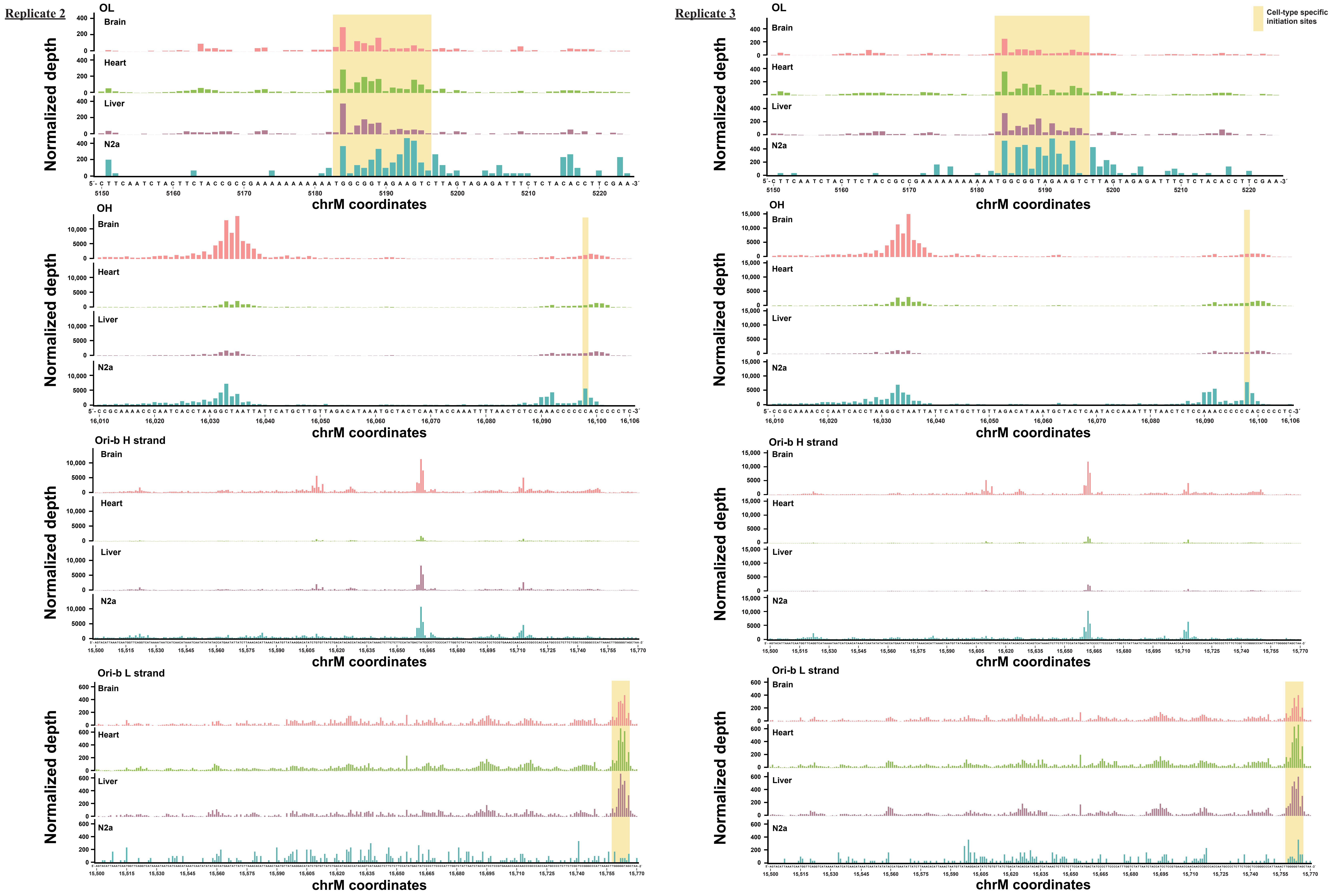

Fig.S3. Detailed views of the 5'-end signals in the mouse mitochondrial replication origins OL, OH and Ori-b — the second and third biological replicates. Normalized depths of the SSiNGLe-5'ES signal (Y-axes) derived from the L (OL and Ori-b) or H (OH and Ori-b) strands of the mitochondrial genome in the mouse brain, heart, and liver tissues and the N2a cell line are shown. Cell-type specific initiation sites are highlighted in yellow. The X-axes represent genomic coordinates. The corresponding results from the first biological replicate are shown in Fig.5.

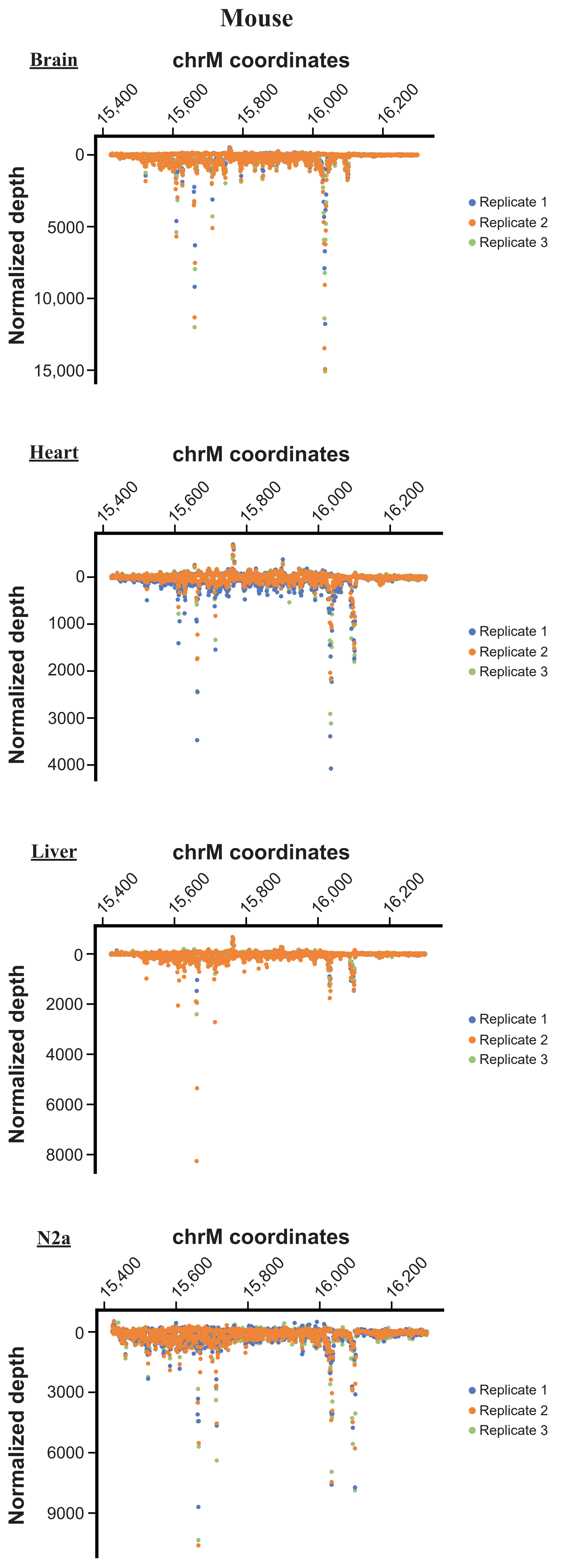

Fig.S4. SSiNGLe-5'ES profile in the mouse NCR. Normalized depths of the SSiNGLe-5'ES signal (Y-axes) from each of the three biological replicates for each mouse tissue and N2a cell line are shown. The SSiNGLe-5'ES signal for the L strand is shown above the "0" line, while the signal for the H strand is shown below.

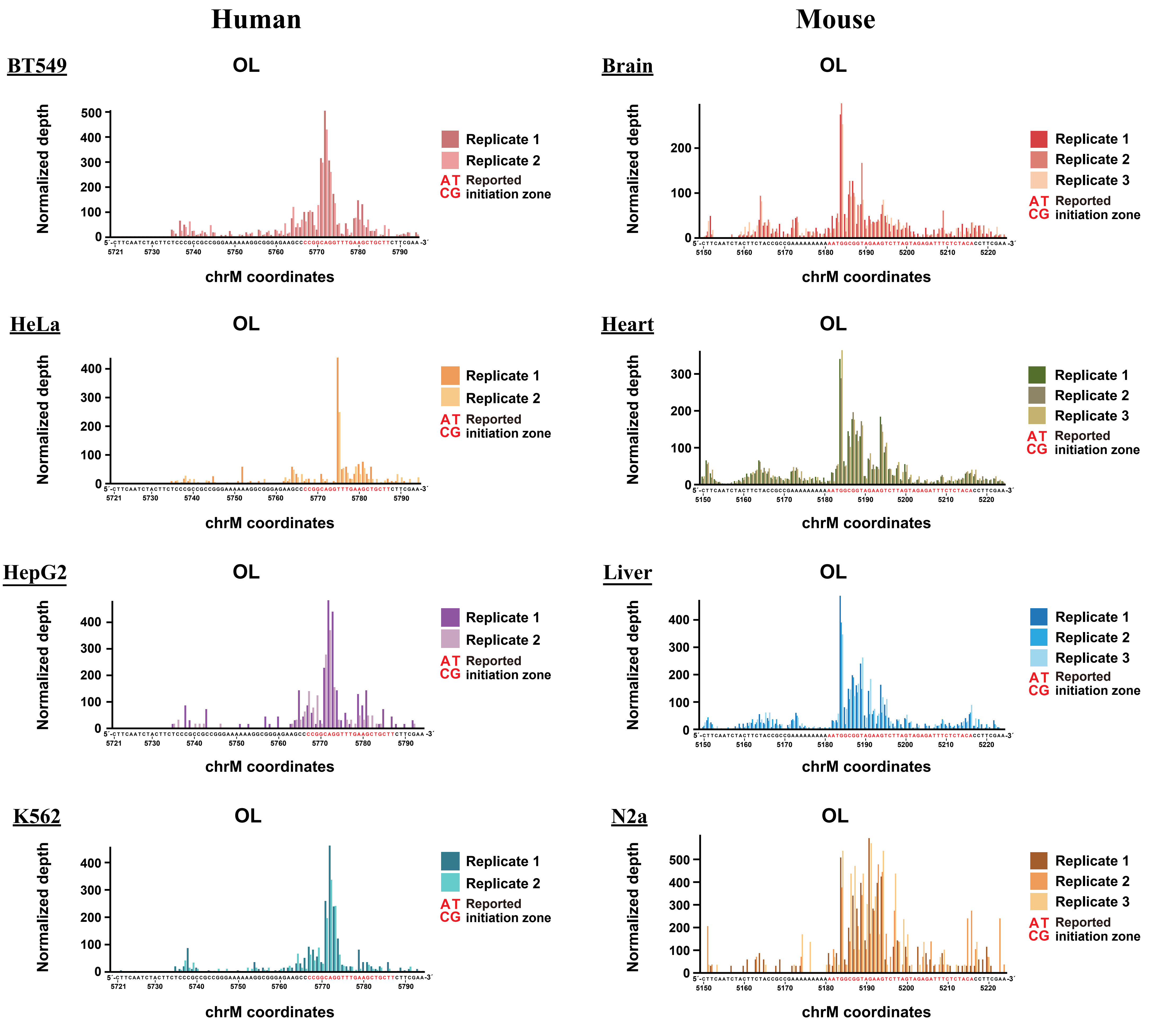

Fig.S5. Detailed views of the 5'-end signals in OL in the human and mouse samples in all biological replicates. The normalized depths of the SSiNGLe-5'ES signal (Y-axes) from the L strand of the human and mouse mitochondrial genomes are shown. Red letters indicate reported OL initiation zones. The X-axes represent genomic coordinates.
